# Hyperprolactinemia-inducing antipsychotics increase breast cancer risk by activating JAK-STAT5 in precancerous lesions

**DOI:** 10.1186/s13058-018-0969-z

**Published:** 2018-05-19

**Authors:** A. N. Johnston, W. Bu, S. Hein, S. Garcia, L. Camacho, L. Xue, L. Qin, C. Nagi, S. G. Hilsenbeck, J. Kapali, K. Podsypanina, J. Nangia, Y. Li

**Affiliations:** 1 Translational Biology and Molecular Medicine, Baylor College of Medicine, Houston, TX 77030 USA; 2 Lester and Sue Smith Breast Center, Baylor College of Medicine, Baylor College of Medicine, Houston, TX 77030 USA; 3 Dan L. Duncan Cancer Center, Baylor College of Medicine, Houston, TX 77030 USA; 4 Molecular and Cellular Biology, Baylor College of Medicine, Houston, TX 77030 USA; 5 SMART PREP Program, Baylor College of Medicine, One Baylor Plaza, Houston, TX 77030 USA; 6 Institut Curie, PSL Research University, CNRS, UMR3664, Equipe Labellisée Ligue contre le Cancer, F-75005 Paris, France; 7 Sorbonne Universités, UPMC Université Paris 06, CNRS, UMR3664, F-75005 Paris, France; 8 Molecular Virology and Microbiology, Baylor College of Medicine, One Baylor Plaza, Houston, TX 77030 USA

**Keywords:** Cancer, Breast cancer, Antipsychotics, Neuroleptics, Prolactin, STAT5, JAK, Ruxolitinib, Mammary gland

## Abstract

**Background:**

Psychiatric medications are widely prescribed in the USA. Many antipsychotics cause serum hyperprolactinemia as an adverse side effect; prolactin-Janus kinase 2 (JAK2)-signal transducer and activator of transcription 5 (STAT5) signaling both induces cell differentiation and suppresses apoptosis. It is controversial whether these antipsychotics increase breast cancer risk.

**Methods:**

We investigated the impact of several antipsychotics on mammary tumorigenesis initiated by retrovirus-mediated delivery of either *ErbB2* or *HRas* or by transgenic expression of *Wnt-1*.

**Results:**

We found that the two hyperprolactinemia-inducing antipsychotics, risperidone and pimozide, prompted precancerous lesions to progress to cancer while aripiprazole, which did not cause hyperprolactinemia, did not. We observed that risperidone and pimozide (but not aripiprazole) caused precancerous cells to activate STAT5 and suppress apoptosis while exerting no impact on proliferation. Importantly, we demonstrated that these effects of antipsychotics on early lesions required the *STAT5* gene function. Furthermore, we showed that only two-week treatment of mice with ruxolitinib, a JAK1/2 inhibitor, blocked STAT5 activation, restored apoptosis, and prevented early lesion progression.

**Conclusions:**

Hyperprolactinemia-inducing antipsychotics instigate precancerous cells to progress to cancer via JAK/STAT5 to suppress the apoptosis anticancer barrier, and these cancer-promoting effects can be prevented by prophylactic anti-JAK/STAT5 treatment. This preclinical work exposes a potential breast cancer risk from hyperprolactinemia-inducing antipsychotics in certain patients and suggests a chemoprevention regime that is relatively easy to implement compared to the standard 5-year anti-estrogenic treatment in women who have or likely have already developed precancerous lesions while also requiring hyperprolactinemia-inducing antipsychotics.

**Electronic supplementary material:**

The online version of this article (10.1186/s13058-018-0969-z) contains supplementary material, which is available to authorized users.

## Background

Psychiatric medications are among the top five drugs in sales in the USA [1]. Both typical (class 1) and atypical (class 2) antipsychotics (also known as neuroleptics) act by antagonizing dopamine and thus blocking post-synaptic dopamine D2 receptors in the pituitary gland; atypical antipsychotics additionally suppress serotonin receptors [2]. Dopaminergic receptors typically suppress prolactin (PRL) production and secretion; thus, a multitude of antipsychotics are associated with elevated serum PRL [2]. A retrospective study of 422 psychiatric patients found that antipsychotic therapy was strongly associated with hyperprolactinemia [3], and that serum PRL levels were affected in a dose-dependent manner [4–6].

PRL binds to its receptor PRLR to activate Janus kinase 2 (JAK2). JAK2 then phosphorylates and activates the signal transducer and activator of transcription 5 (STAT5). Once phosphorylated, STAT5 forms homodimers or heterodimerizes with another STAT family member, translocates to the nucleus, and transactivates its targets, which regulate alveolar differentiation and milk production and proliferation and apoptosis [7]. PRL-JAK2-STAT5 signaling is highly activated during late pregnancy and lactation, and is required for alveolar expansion and milk production [7]. Transgenic or retrovirus-mediated expression of constitutively activated STAT5 in normal mammary epithelia in nulliparous mice causes alveolar differentiation and milk production [8, 9]. Hyperprolactinemia associated with the use of antipsychotics of both classes often causes mammary swelling and lactation that are not associated with pregnancy [10]. Mammary cell differentiation caused by PRL-PRLR-JAK2-STAT5 signaling is a mechanism by which an early-age pregnancy reduces breast cancer risk [11]. However, we have also reported that STAT5 activation in preexistent precancerous lesions in mice instigates accelerated progression to cancer via suppression of the apoptosis anticancer barrier [11]. This finding provides an explanation for increased breast cancer risk associated with a late-age pregnancy when early lesions may have already formed. Activated forms of JAK2 and STAT5 have been reported in human early breast lesions and cancer [7, 12–16] and in other human cancers [7, 17].

While it explains the dichotomous effects of early versus late-age pregnancy on breast cancer risk, the dual-role of PRL-JAK2-STAT5 in both promoting normal cell differentiation and suppressing the anticancer barrier in precancerous cells also predicts that hyperprolactinemia-inducing antipsychotics may have a similar dichotomous impact on breast tumorigenesis – reducing breast cancer risk when taken at a young age but increasing breast cancer risk if started at an older age or when early lesions have already been diagnosed. However, when started at an early age, this type of medication is usually taken for decades or for lifetime [18], likely leading to protection against breast cancer earlier on but increased risk later in life. Therefore, epidemiological studies of breast cancer risk in patients on antipsychotics must consider hyperprolactinemia, starting age and length of treatment, precancerous lesion status, and multiple other confounding factors such as obesity and poor health status [2, 19–23]. The limited work in this area has not stratified patients to consider all of these variables. Not surprisingly, these studies have resulted in inconclusive or contradictory reports [19, 24–31], although a few studies have detected a significant increase in breast cancer in women who were prescribed dopamine antagonists compared to age-matched controls who were not prescribed antipsychotics [32, 33]. Data from well-controlled laboratory studies may provide the experimental foundation for sophisticated epidemiological studies that will involve multiple patient registries and are stringently controlled. Importantly, laboratory studies may especially expose potential cancer risk of administering hyperprolactinemia-inducing antipsychotics in patients who may have already developed precancerous lesions. However, there have been no significant laboratory studies to investigate the influence of antipsychotics on breast cancer risk. Here, we report that in mouse models that closely mimic human breast cancer initiation, hyperprolactinemia-inducing antipsychotics accelerate early lesion progression to cancer via activation of JAK-STAT5 signaling to suppress the apoptosis anticancer barrier. These findings highlight the potential risk associated with the use of hyperprolactinemia-inducing antipsychotics in women at risk of breast cancer and urgently calls for epidemiological studies specifically designed to examine breast cancer risk in women who have already developed precancerous lesions while also requiring hyperprolactinemia-inducing antipsychotics.

## Methods

### Experimental animals

The mouse mammary tumour virus promoter (MMTV-*tva*) (MA line) and STAT5a^−/−^ mice used in this study are on a Friend virus B (FVB) genetic background and have been previously reported [34, 35]. Briefly, lesion initiation is achieved by intraductal injection of a Rous sarcoma virus-based vector - replication-competent avian sarcoma (RCAS) - to deliver an oncogene into a minute subset of mammary epithelial cells in an otherwise normally developed mammary gland [34]. This allows cancer to initiate in a “field” of normal mammary cells in a normal mammary gland as human breast cancer usually initiates and evolves [34]. This subset of mammary epithelial cells was made susceptible to RCAS infection by transgenic expression of the gene encoding the RCAS receptor TVA from the MMTV promoter (MMTV-*tva*) [34]. STAT5a^−/−^ mice have been previously described [35]; the *STAT5a* knockout mice on the FVB background have normal mammary development unlike those on the 129 background [11, 35]. All animals were handled according to the animal protocol approved by Baylor College of Medicine (BCM) Institutional Animal Care and Use Committee (IACUC).

### Early lesion and tumor studies

RCAS virus was prepared as previously described [34, 36] and was intraductally injected into MMTV-*tva* mice at 10 weeks of age. Five days later they were randomized and treated with either a drug or diluent for 2 weeks (early lesion studies) or until euthanasia (tumor study). Mice in the tumor latency study were palpated thrice weekly and tumor size was recorded. When tumors reached 2.0 cm in diameter, cumulatively, the mice were euthanized. Tumor-free mice were euthanized 12 months post injection.

### Drug treatments

Pimozide (cat. no. P1793; Sigma-Aldrich) was intraperitoneally (IP) administered daily at 5 mg/kg. Risperidone (cat. no. 1604654; Sigma-Aldrich) was delivered IP daily (3 mg/kg) for 2 weeks (early lesion study) or in drinking water (1.56 mg/l) until euthanasia (tumor latency study), resulting in the same daily dose based on the calculation previously reported [37]. Aripiprazole (cat. no. SML0935; Sigma-Aldrich) was delivered via IP injection in a daily dose of 3 mg/kg for 2 weeks, and clomipramine (cat. no. 1140247; Sigma-Aldrich) was delivered in drinking water (190 mg/l), resulting in a daily dose of 28 mg/kg. All drugs were diluted in dimethyl sulfoxide (DMSO) to the appropriate concentrations. Both ruxolitinib and control chow was provided by Incyte Corp. Ruxolitinib chow was packaged in a pre-determined measurement of 2000 mg/kg chow; mice were allowed to free-feed for the duration of the study.

### Serum PRL

Serum PRL was determined using the Sigma-Aldrich Mouse Prolactin ELISA kit (RAB0408) using the manufacturer’s protocol.

### Immunostaining and microscopy

Immunohistochemistry analysis (IHC) and immunofluorescence (IF) were performed as previously described [9, 11, 34]. MOM and vectastain Elite ABC rabbit kits (cat.no. PK-2200 and PK-6101; Vector Laboratories) were used according to the manufacturer’s protocols. Primary antibodies used included mouse monoclonal antibodies against HA (1:250; cat.no.901503; Covance) and BCL-xL (1:50; cat.no. K1308; Santa Cruz) and rabbit antibodies against pSTAT5 1:300; cat.no. 9359 L; Cell Signaling), cleaved caspase 3 (1:300; cat.no. Asp175; Cell Signaling), and Ki67 (1:300; cat.no. MIB-1; Lycra). Secondary antibodies for IF were Alexa Fluor 568 goat-anti-rabbit, and Alexa 488 goat-anti-mouse. Nuclei were counterstained with 4′-6-diamidino-2-phenylindole (DAPI)-containing mounting medium and hematoxylin, respectively, for IF and IHC. TUNEL assay was performed using the ApopTag Red in situ TUNEL detection Kit (Chemicon, S7165). Bright-field images were captured using a Leica DMLB microscope. IF images were captured using the Zeiss Axiskop2 plus microscope.

### Quantification of stained sections

For quantification of cells stained for a marker, 10 random fields of early lesions in each mammary gland were captured, and both positively stained cells and the total number of cells in the lesion as identified by DAPI or hematoxylin staining were counted to determine the percentage of positivity. ImageJ software was used for counting cells and determining lesion size. The total numbers of cells in IF images were counted using a semi-automotive program that has been previously described [38]. Fixed thresholds were set to analyze both experimental and control mammary glands.

### Lung metastasis study

Lung metastases were detected by the quantitative-PCR (qPCR) method using a set of primers specific for the RCAS provirus (CTTCCCTGCCGCTTCC; FWD: AGCCGCCTCAAGTCATGATG; GCTCTTTCCAATGTACCGATAACCT). DNA was extracted from the largest, left-most lobe of the lung. A mammary tumor induced by RCAS-caErbB2 was used as positive control, and a lung from a FVB mouse without virus injection was used as negative control. The relative amounts of the RCAS provirus with respect to the endogenous gene β-actin were determined using the 7500 Fast System software provided by Applied Biosystems.

### Statistical analysis

All numbers in this study are reported as medians and interquartile ranges in the format median (IQR). Statistical analyses of quantification of stained sections were performed using analysis of variance (ANOVA) or the Mann-Whitney test. In cases where data were distributed normally, Student’s *t* test for independent samples with Holm’s correction for multiple comparisons was used. Tumor-free survival analysis was performed using the generalized Gehan-Wilcoxon test with Rho = 1, and Kaplan-Meier survival curves. All tumor-free survival analyses were performed in R with the survival package using R commander interface. All other graphs were generated using Prism software. Each dot in the dot plots generated for this study represents one mouse.

## Results

### Treatment with hyperprolactinemia-inducing antipsychotics accelerates tumorigenesis from breast cancer cells with an oncogenic mutation

**Fig. 1:**
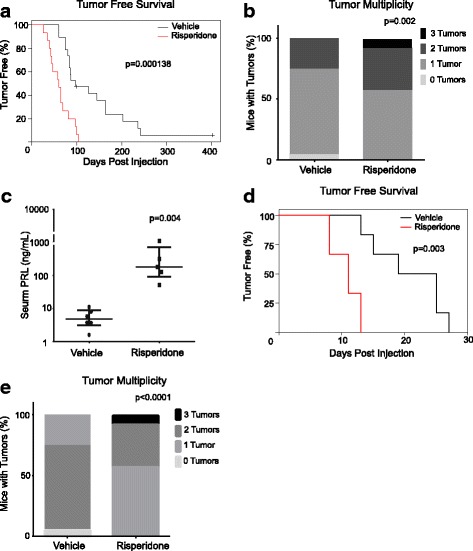
Risperidone promotes carcinogenesis initiated by ca*ErbB2* and *HrasQ61L*. **a** Kaplan-Meier tumor-free survival curve of mice infected by replication-competent avian sarcoma (RCAS)-caErbB2. The *p* value was determined by the generalized Gehan-Wilcoxon test with Rho = 1. **b** Tumor multiplicity. The chi square test was for used comparison. **c** Serum prolactin (PRL) levels. The Mann-Whitney test was used to determine the *p* values. Each dot in this plot represents one mouse. **d** Kaplan-Meier tumor-free survival curve of mice infected by RCAS-HRasQ61L. The *p* value was determined by the generalized Gehan-Wilcoxon test with Rho = 1. **e** Tumor multiplicity. The chi square test was for used comparison

We have reported a mouse model that closely mimics human breast cancer initiation and is ideally suited for studying hormones and other factors that may impact breast cancer risk [9, 11, 34, 38–42]. Tumor initiation is achieved by intraductal injection of a Rous sarcoma virus-based vector, RCAS, to deliver an oncogene into a small subset of mammary epithelial cells (< 0.3% of the mammary gland) in a normally developed mammary gland so that cancer initiates in a “field” of normal mammary cells in a normal mammary gland as most human breast cancers initiate and evolve [34]. This subset of mammary epithelial cells was made susceptible to RCAS infection by transgenic expression of the gene encoding the RCAS receptor TVA from the MMTV promoter (MMTV-*tva*) [34]. The transgenic avian *tva* is only required for the initial virus infection; the virus does not replicate in mammalian cells. Additionally, the oncogene is transcriptionally controlled by the proviral RCAS long terminal repeat (LTR); it is constitutively active and is not influenced by the presence of reproductive hormones such as prolactin [39, 43]. Using this method, we explored the effect of several antipsychotics on mammary cancer development from preexisting early lesions. We injected 10 week-old MMTV-*tva* mice intraductally with RCAS-caErbB2 to infect approximately 0.3% of the luminal epithelial cells [34, 44]. RCAS-caErbB2 expresses a constitutively active form of rat *ErbB2* (*HER2/Neu)*; *ErbB2* is amplified/mutated in 20–25% of human breast cancers [45]. Five days following injection, mice were randomized for treatment with risperidone (3 mg/kg daily), a commonly prescribed class 2, “atypical” antipsychotic that is known to cause hyperprolactinemia [2], or with vehicle. Introduction of the oncogene took place before drug treatment so as to specifically investigate antipsychotic effects on preexisting precancerous early lesions rather than the overall risk that antipsychotics may pose on the normal mammary epithelia. Mice were continually treated with either risperidone or the diluent control in drinking water for the duration of the study. While vehicle-treated mice developed tumors with a median latency of 112 days, the risperidone cohort developed tumors with a median latency of only 59 days (*p* = 0.000138; Fig. 1a). Additionally, the risperidone-treated cohort had a greater tumor multiplicity than the control (*p* = 0.002; Fig. 1b). When the tumor size reached 2.0 cm in diameter, mice were euthanized. As expected, serum PRL levels were significantly increased in the risperidone cohort (*p* = 0.004; Fig. 1c). Tumors from both cohorts were high-grade, poorly differentiated, and highly mitotic with areas of necrosis. Many of the tumor cells were highly pleomorphic with large nuclei, often with metaplastic features. These tumors extensively invaded the surrounding fibroadipose tissue, skeletal muscle, and nerves (Additional file 1: Figure S1A). Likewise, incidence of pulmonary metastasis was similar in the two cohorts of mice based on qPCR analysis of the RCAS-proviral load (*p* = 0.753; Additional file 1: Figure S1B). Therefore, we conclude that treatment with risperidone accelerates tumorigenesis and increases tumor multiplicity in mice with preexisting precancerous mammary lesions, while not influencing the grade, aggressiveness, or metastatic potential of the resulting tumors.

To investigate whether risperidone also accelerated tumorigenesis initiated by other oncogenic events, we injected MMTV-*tva* mice (n = 25), at 10 weeks of age, with RCAS carrying an activated form of *HRas*, *HRasQ61L* (RCAS-HRasQ61L). *RAS* genes are known to be amplified/overexpressed/mutated in a subset of human breast cancer, and their protein products often activated [46–49]. Five days following injection, mice were continually treated with risperidone or vehicle in their drinking water. While vehicle-treated mice developed tumors with a median latency of 25 days, the risperidone cohort developed tumors with a median latency of only 11 days (*p* = 0.003; Fig. 1d). Additionally, the risperidone-treated cohort had a higher tumor multiplicity than the control (*p* < 0.0001; Fig. 1e). Taken together, these data suggest that risperidone stimulates tumorigenesis initiated by multiple oncogenic events.

**Fig. 2:**
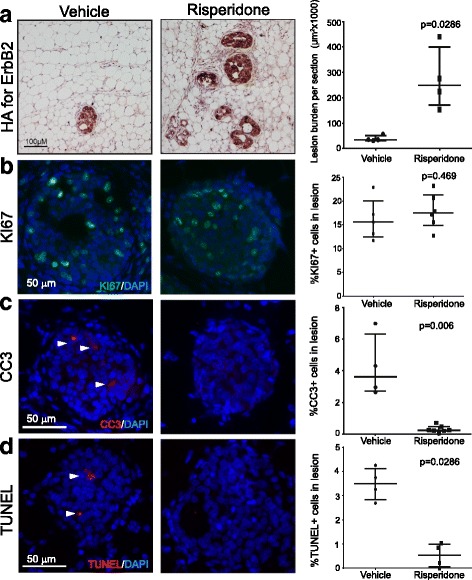
Risperidone increases early lesion burden and lowers the level of apoptosis**. a** Immunohistochemistry analysis and the accompanying dot plot for the HA tag on replication-competent avian sarcoma (RCAS)-caErbB2 provirus. **b** Immunofluorescence for Ki67 and the accompanying dot plot. **c** and **d** Immunofluorescence staining for cleaved caspase 3 (**c**) and TUNEL assay (**d**) with the accompanying dot plots. The *p* values were determined by the Mann-Whitney test. Each dot in these plots represents one mouse

We next determined if this tumorigenic acceleration was due to antipsychotic effects on early lesion development. Here we used RCAS-caErbB2-infected mice and treated them with risperidone or vehicle for only 2 weeks. Early lesions were defined as any hyperplastic ductal foci comprised of three or more layers of epithelial cells stained positively for the provirus-encoded oncogene product HA tag. Risperidone-treated mice had more early lesions and higher early lesion burden than the vehicle control cohort (Fig. 2a). Therefore, we conclude that risperidone promotes early lesion progression.

To test whether the above-observed risperidone effect on mammary early lesions is broadly applicable across different subtypes of breast cancer, we administered risperidone or vehicle to mice transgenic for MMTV-*Wnt1* [50], which develops basal-like tumors and some estrogen receptor (ER)-positive tumors [51–53]. Mice treated with risperidone for 2 weeks developed extensive ductal branching and many more and larger early lesions compared to vehicle-treated mice (Additional file 2: Figure S2A-2B). Taken together, these data suggest that risperidone stimulates the progression of early lesions that are the precursor to multiple breast cancer subtypes.

To determine the underlying mechanisms by which risperidone spurred early lesion expansion, we first compared the precancerous cell proliferation in these two cohorts of mice injected with RCAS-caErbB2. Ki67 staining detected approximately 20% of positive cells in both sets of early lesions (*p* = 0.469; Fig. 2b), suggesting that proliferation did not play a significant role in risperidone acceleration of early lesion development. Besides cell proliferation, evasion of apoptosis serves a key role in the progression of precancerous early lesions to cancer [54, 55]. We have reported that apoptosis was rapidly activated in mammary cells following *ErbB2* activation to provide a barrier to cancer [11, 42, 44]. Both cleaved caspase 3 (CC3) and TUNEL detected robust apoptosis (3.6% (2.7–6.3%) and 3.5% (2.8–4.1%), respectively) in early lesions in vehicle-treated mice, as expected; however, these apoptosis levels were diminished in the risperidone cohort (0.2% (0.07–0.5%), *p* = 0.006 and 0.5% (0.05–0.9%), *p* = 0.0286, respectively) (Fig. 2c-d). These results demonstrate that treatment with risperidone allows precancerous cells to suppress the apoptotic anticancer barrier to increase early lesion burden.

To test whether other hyperprolactinemia-inducing antipsychotics also instigate early lesion progression, we treated a separate cohort of early lesion-bearing mice with pimozide, which is a “typical”, class 1 antipsychotic that is also known to induce hyperprolactinemia [10]. As expected, this antipsychotic also led to hyperprolactinemia in these mice (*p* = 0.007; Additional file 3: Figure S3A). Like risperidone, pimozide (5 mg/kg daily) for 2 weeks increased early lesion numbers (from 22 (12.8–31) to 64 (42.5–98), *p* = 0.004) and led to a greater early lesion burden (Additional file 4: Figure S4A). While not affecting cell proliferation (Additional file 4: Figure S4B), pimozide suppressed apoptosis in early lesions based on both CC3 and TUNEL (*p* = 0.0002 and *p* = 0.0007, respectively; Additional file 4: Figure S4C-3D). Taken together, these data indicate that hyperprolactinemia-inducing antipsychotics dismantle the apoptosis anticancer barrier in early lesions and instigate their progression to cancer.

To investigate whether antipsychotics that do not cause hyperprolactinemia have the potential to accelerate early lesion progression, we tested aripiprazole, a widely prescribed class 2, “atypical” antipsychotic that does not elevate prolactin levels but is otherwise mechanistically similar to risperidone [2]. Two weeks of aripiprazole (28 mg/kg daily) did not elevate serum prolactin levels (Additional file 5: Figure S5A), and failed to increase the load of RCAS-caErbB2-initiated early lesions (Additional file 5: Figure S5B). Likewise, this drug did not affect cell proliferation (Additional file 5: Figure S5C) or apoptosis as tested by TUNEL (Additional file 5: Figure S5D). In addition, clomipramine, a commonly prescribed antidepressant that did not cause hyperprolactinemia in our mouse model (*p* = 0.151; Additional file 5: Figure S5E) also failed to increase early lesion burden (*p* = 0.687; Additional file 5: Figure S5F). Together, we conclude that hyperprolactinemia-inducing antipsychotics cause preexisting early lesion to suppress the apoptosis anticancer barrier and to accelerate progression to cancer; these cancer-promoting effects are associated with hyperprolactinemia.

### Hyperprolactinemia-inducing antipsychotics activate STAT5

**Fig. 3:**
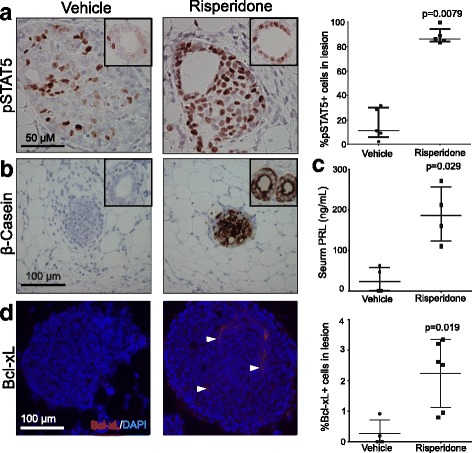
Risperidone treatment increases signal transducer and activator of transcription 5 (STAT5) activity. **a** Immunohistochemistry staining for pSTAT5 in early precancerous lesions and in normal ducts (inset) and the accompany dot plot. **b** Immunohistochemistry analysis of the downstream effector of STAT5, β-casein, in early lesions and normal ducts (inset). **c** Serum prolactin (PRL) levels. **d** Immunofluorescence and the accompanying dot plot for Bcl-xL. The *p* values were determined by the Mann-Whitney test. Each dot in these plots represents one mouse

PRL is a key hormone released during pregnancy and lactation, and activates STAT5 via its receptor PRLR and the receptor-associated JAK2 [7]. Forced or pregnancy-associated JAK2/STAT5 activation lowers the apoptosis anticancer barrier in preexisting early lesions and advances the progression to cancer [11]. To understand the underlying mechanism by which hyperprolactinemia-inducing antipsychotics suppress the apoptosis anticancer barrier and increase breast cancer risk, we asked whether treatment with risperidone activates the STAT5 signaling pathway. In vehicle-treated mice bearing early lesions initiated by RCAS-caErbB2, pSTAT5+ cells were detected in 11% (5.7–30.3%) of precancerous cells, a level similar to those previously reported [11]; however, 2 weeks of risperidone treatment increased their population size to 86.4% (84.2–94.3%) (*p* = 0.0079; Fig. 3a). Induction of STAT5 activity was also detected in normal ducts that did not gain ca*ErbB2* (Fig. 3a). We confirmed that serum PRL levels were significantly increased in the risperidone cohort (*p* = 0.029; Fig. 3c). *Bcl-xL* and *β-cas*ein are genes that are transactivated by STAT5, and their gene products contribute to cell survival and alveolar differentiation, respectively [56, 57]. As expected, β-casein was induced in early lesions and in normal ducts (Fig. 3b). Likewise, Bcl-xL was induced in early lesions (Fig. 3d). Additionally, we found that pSTAT5 levels were elevated in lesions of the risperidone-treated MMTV-Wnt1 transgenic mice compared to the vehicle-treated control cohort (Additional file 2: Figure S2C).

Next, we tested whether pSTAT5 and its transcriptional target β-casein were also upregulated by pimozide. While 7.3% (3.4–14.6%) of cells in the early lesions of vehicle-treated mice were pSTAT5+, 58% (50–89%) of cells in the early lesions of pimozide-treated mice were pSTAT5+ (*p* = 0.0007; Additional file 3: Figure S3B). β-casein was also induced following pimozide administration (Additional file 3: Figure S3C). Of note, pimozide has been reported to block STAT5 activation in cultured cells [58, 59], but this potential direct effect on STAT5 was overridden in vivo by hyperprolactinemia-induced PRL signaling. To further test the association between hyperprolactinemia and activation of STAT5, we also stained for pSTAT5 in early lesions in mice treated with aripiprazole, which did not increase PRL. No evidence of STAT5 activation was detected (Additional file 3: Figure S3D). Together, these data suggest that hyperprolactinemia-inducing antipsychotics activate STAT5 and its transcriptional targets to suppress the apoptosis-anticancer barrier in preexisting precancerous cells, while simultaneously inducing alveolar differentiation in both early lesions and normal ducts.

### STAT5a is required for antipsychotic promotion of mammary tumorigenesis

**Fig. 4:**
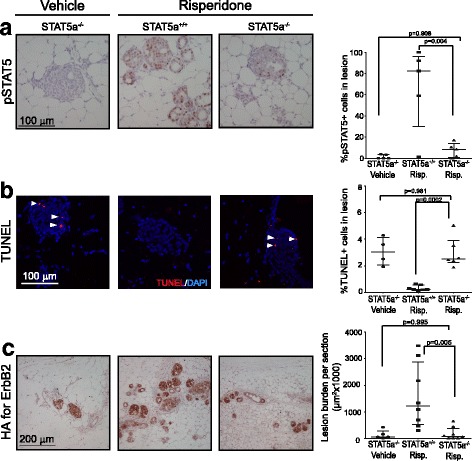
Genetic ablation of signal transducer and activator of transcription 5 (*STAT5*)*a* dismantles the effects of risperidone on early lesions. **a** pSTAT5 immunohistochemistry analysis and the accompanying dot plot. **b** TUNEL assay and the accompanying dot plot. **c** Immunohistochemistry analysis and the accompanying dot plot for the HA tag on replication-competent avian sarcoma (RCAS)-*caErbB2* provirus. The *p* values were determined by analysis of variance. Each dot in these plots represents one mouse

PRL-PRLR signaling activates STAT5 and possibly several other pathways including extracellular signal-related kinase (Erk) and protein kinase B (Akt) [60]. We tested whether the gene encoding STAT5a, the predominant form of STAT5 in the mammary gland and tumorigenesis [35, 61], is required for the above-observed effects of risperidone on early lesion progression, to investigate whether STAT5 activation is the crucial factor mediating antipsychotic stimulation of carcinogenesis. We utilized *STAT5a*^−/−^ mice on the FVB background as *STAT5a* ablation on this background does not significantly impair normal mammary development and lactogenesis [11]. These *STAT5a* knockout mice were bred to MMTV-*tva* mice, infected with RCAS-caErBb2, and 5 days later, continually treated with either risperidone or vehicle control for 2 weeks. *STAT5a*^+/+^/MMTV-*tva* mice infected with RCAS-caErbB2 were also treated with risperidone for 2 weeks for comparison. Risperidone treatment failed to activate STAT5 in *STAT5a*^−/−^ mice – the percentage of pSTAT5+ cells was comparable to that in the vehicle-treated mice (*p* = 0.908) and much lower than that in *STAT5a* wild-type mice treated with risperidone (*p* = 0.004; Fig. 4a). The residual levels of pSTAT5 in *STAT5a*^−/−^ mice were likely due to the minor player pSTAT5b. Additionally, β-casein, a transcriptional target of STAT5, was elevated in the risperidone-treated *STAT5a* wild-type mice, but severely diminished in the *STAT5a* knockout risperidone-treated group (Additional file 6: Figure S6). Next, we asked if *STAT5a*-knockout-induced diminishment of STAT5 activity restored apoptosis in early lesions in the risperidone cohort. In comparison to the wild-type mice treated with risperidone, the *STAT5a*^−/−^ mice treated with risperidone had significantly elevated levels of apoptosis as measured by TUNEL (*p* = 0.0002; Fig. 4b), which were comparable to those in the vehicle-treated *STAT5a*^−/−^ mice (*p* = 0.981; Fig. 4b). This finding indicates that *STAT5a* plays a pivotal role in the effects of risperidone on evading the apoptotic anticancer barrier. Next, we examined whether STAT5 activity reduction also led to a lower early lesion burden in risperidone-treated mice. Indeed, *STAT5a*^−/−^ mice treated with risperidone had significantly lower levels of the early lesion burden than the *STAT5a*^+/+^ mice treated with risperidone (*p* = 0.005; Fig. 4c), and further, these low levels were comparable to the levels in the vehicle control *STAT5a*^−/−^ mice (*p* = 0.995; Fig. 4c). Taken together, these results demonstrate that STAT5 activity is responsible for evading the apoptosis anticancer barrier in early lesions and for instigating early lesion progression.

### Prophylactic treatment with an inhibitor of the JAK/STAT signaling pathway restores the apoptosis anticancer barrier in early lesions and decelerates their progression

**Fig. 5:**
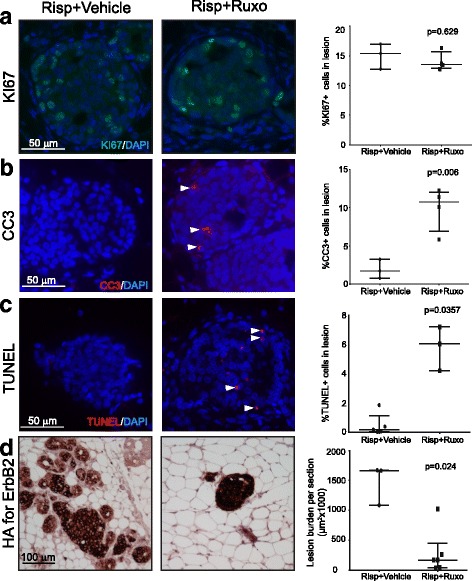
Ruxolitinib (Ruxo) treatment restore the apoptosis anticancer barrier and blocks early lesion expansion. **a** Immunofluorescence staining for Ki67 and the resulting dot plot. **b** and **c** Immunofluorescence staining for cleaved caspase 3 (**b**) and TUNEL assay (**c**) with the accompanying dot plots. **d** Immunohistochemistry analysis and the accompanying dot plot for the HA tag on RCAS-ca*ErbB2* provirus. The *p* values were determined by the Mann-Whitney test. Each dot in these plots represents one mouse. Risp, risperidone

Many older women on hyperprolactinemia-inducing antipsychotics may have already accumulated early lesions and subsequently may be at increased risk of breast cancer due to family history, older age, or other reasons. Our aforementioned findings suggest that these antipsychotics likely also stimulate the progression of the early lesions in these high-risk women. For these women, it may be advisable to switch to another antipsychotic that does not cause hyperprolactinemia; however, switching to another drug is often difficult for fear of relapse and/or withdrawal effects. Consequently, it is important to identify effective breast cancer preventive strategies in high-risk women who need to take these types of antipsychotics. There are currently only a few US Food and Drug Administrtion (FDA)-approved drugs for breast cancer chemoprevention, all of which antagonize estrogen signaling [62]. As these drugs require 5 years of continuous treatment to lower breast cancer risk by 50%, do not prevent estrogen receptor (ER)-negative cancer, and can have significant side effects, they are not widely used for prevention [62]. However, we have previously reported that short-term suppression of either pSTAT5 or JAK1/2 activity can restore the apoptosis anticancer barrier and reduce mammary tumor risk in mouse models [11]. Ruxolitinib is an FDA-approved small molecule inhibitor of JAK1/2 for the treatment of myelofibrosis and polycythemia vera; it has minimal significant side effects following short-term use in healthy individuals [63–66]. Therefore, we asked whether short-term ruxolitinib treatment could prevent mammary tumors in early lesion-bearing mice on risperidone. Here, ruxolitinib-supplemented or control chow was fed to risperidone-treated mice bearing early lesions initiated by RCAS-caErbB2. After 2 weeks of treatment, serum PRL levels in both cohorts remained elevated, as expected (*p* = 0.98; Additional file 7: Figure S7A); however, ruxolitinib significantly lowered the percentages of pSTAT5+ cells in early lesions (*p* < 0.0001; Additional file 7: Figure S7B), indicating that risperidone-induced activation of STAT5 depends on JAK1/2 activity. Ruxolitinib did not affect precancerous cell proliferation based on Ki67 (*p* = 0.629; Fig. 5a), as expected from non-detectable impact on precancerous cell proliferation by risperidone; however, mice fed with ruxolitinib chow had restored apoptosis in early lesions as measured by CC3 and TUNEL (*p* = 0.006 and 0.0357, respectively; Fig. 5b-c), reaching/surpassing the levels detected in early lesions in mice not treated with any antipsychotic (Fig. 2c-d). Importantly, these mice had a much lower early lesion burden than the mice on the control chow (*p* = 0.024; Fig. 5d). Furthermore, we investigated whether ruxolitinib could prevent/delay tumor appearance. Here, we injected MMTV-*tva* mice (n = 14), 10 weeks of age, with RCAS-HrasQ61L virus. Five days later, we randomized the mice into two groups for risperidone water plus ruxolitinib-supplemented chow or for risperidone water plus control chow. While the control-chow-treated mice on risperidone developed tumors with a median latency of 9 days, the ruxolitinib-chow cohort on risperidone developed tumors with an extended median latency of 13 days (*p* = 0.008; Additional file 7: Figure S7C). These data further confirmed the importance of PRL-stimulated JAK-STAT5 signaling in lowering the apoptosis anticancer barrier and in increasing early lesion burden and progression in mice on risperidone. Taken together, short-term treatment with ruxolitinib restores apoptosis in early lesions, reduces early lesion burden, and lowers mammary tumor risk in mice on hyperprolactinemia-inducing antipsychotics; therefore, these data suggest that prophylactic treatment with ruxolitinib may lower breast cancer risk in high-risk women who are on these antipsychotics.

## Discussion

Using three different mouse models of breast cancer, we demonstrated that hyperprolactinemia-inducing antipsychotics cause preexisting premalignant lesions in the mammary gland to lower the apoptosis anticancer barrier and to accelerate the progression to cancer. We further demonstrated that this cancer-instigating effect is via upregulation of the STAT5 signaling pathway, which is known to transactivate anti-apoptosis genes including *Bcl-xL*. Antipsychotics and other pharmaceuticals of similar classes that do not induce hyperprolactinemia do not activate the STAT5 signaling pathway, nor do they lower the apoptosis barrier; consequently, they do not confer a tumorigenic advantage to precancerous cells. These preclinical findings have important clinical implications. For women who already have preexisting early lesions such as atypical ductal hyperplasia (ADH) or ductal carcinoma in situ (DCIS), taking hyperprolactinemia-inducing antipsychotics for a long term may increase breast cancer risk and should be carefully considered for psychiatric benefits versus breast cancer risk. Unless necessary, perhaps non-hyperprolactinemia-inducing alternatives could be considered as a first-line therapy and should be suggested as a possible substitute in high-risk women who are currently taking hyperprolactinemia-inducing antipsychotics.

Our data also offer an explanation for the contradictory findings in epidemiological studies of association between antipsychotic use and breast cancer risk [19, 24, 27, 28, 30, 31, 67]. First, many of these studies did not differentiate between hyperprolactinemia-inducing antipsychotics and other drugs that do not raise serum PRL levels significantly. Second, and perhaps as equally important, PRL-mediated JAK2-STAT5 signaling has a dichotomous effect on mammary cells. Elevated JAK2-STAT5 signaling can weaken the apoptosis anticancer barrier in precancerous lesions that have already formed in the otherwise normal breast epithelia and thus may potentially increase breast cancer risk [11]. The same signaling pathway can cause the mammary cells to undergo differentiation potentially leading to lower cell proliferation rates and thus may reduce the chance of gaining mutations and consequently breast cancer risk [68]. This dichotomous function of JAK2-STAT5 has been reported by us as a major mechanism to explain the dichotomous effects of pregnancy on breast cancer risk - while an early-age pregnancy protects against breast cancer, a late-age pregnancy increases breast cancer risk [11]. At a young age, the breast epithelia are unlikely to have accumulated mutated cells. Pregnancy at this time induces these normal cells to differentiate. As a result, they become less proliferative and less likely to suffer mutations, and cancer risk is reduced. In contrast, at an older age, mutated cells are more likely to have accumulated. Pregnancy at this time with elevated JAK2-STAT5 activity can cause these precancerous cells to evade the apoptosis anticancer barrier and to evolve into cancer at accelerated speeds. The same may hold true in patients on antipsychotics. While hyperprolactinemia-inducing antipsychotics may increase breast cancer in women who have already gained precancerous cells, they may even lower the cancer risk in women who have not yet accumulated early lesions, such as younger women without any family history of early breast cancer. Consequently, it may not be a surprise that these more general epidemiological studies have generated inconclusive data on antipsychotics and breast cancer risk. Our findings suggest that it is now important to specifically study the association between hyperprolactinemia-inducing antipsychotics and the breast cancer risk in women with preexisting precancerous early lesions.

In order to avoid relapse and withdrawal effects, most patients on antipsychotics receive the same antipsychotic medication for extended periods of time and are sometimes unable to switch to other types of medication, such as those that do not cause hyperprolactinemia. Therefore, if carefully designed epidemiological studies confirm our animal model findings in the human population, prophylactic treatment will then be needed to alleviate the increased breast cancer risk caused by antipsychotics so that patients can still receive the psychiatric care that they need without elevated breast cancer incidence. Currently available chemoprevention drugs require 5 years of continuous treatment, cannot prevent ER-negative tumors that are more difficult to treat, and can have significant side effects [69]. These drawbacks discourage women who are as yet cancer-free from using them. The FDA-approved JAK1/2 inhibitor ruxolitinib has few adverse side effects when used short-term [66, 70, 71]. Even used for only 2 weeks, ruxolitinib was previously found to restore apoptosis in early lesions and to decelerate early lesion progression [11]. In the current study, prophylactic treatment with ruxolitinib induced apoptosis in early lesions and reduced early lesion burden in risperidone-treated mice. This finding predicts that in women with risk factors suggesting the presence of early lesions (ADH and DCIS), short-term or intermittent treatment with ruxolitinib may also reduce precancerous lesion burden and thus negate the elevated breast cancer risk induced by hyperprolactinemia-inducing antipsychotics. Since JAK2-STAT5 signaling plays a crucial role in stimulating early lesion progression even in the absence of antipsychotics [7], such prophylactic treatment may reduce breast cancer risk both associated with and independent of antipsychotic use. An additional benefit of our chemoprevention modality is that it also has the potential to prevent both ER+ and ER- breast cancers, in contrast to current cancer prevention therapies that antagonize estrogen signaling and prevent ER+ breast cancer only [62]. Therefore, this chemoprevention strategy may be highly valuable for women with high breast cancer risk whether they are on antipsychotics or not.

## Conclusions

In conclusion, this mouse model study demonstrates that hyperprolactinemia-inducing antipsychotics activate JAK-STAT5 signaling to lower the apoptosis anticancer barrier in preexisting precancerous early lesions and to incite their progression to cancer. To our knowledge, this is the first study that decisively links antipsychotic use to increased breast cancer risk while also providing a mechanistic insight. Our work also suggests short-term or intermittent ruxolitinib treatment as a potentially effective and more acceptable approach for preventing breast cancer risk in women on these antipsychotics.

## Additional files

Additional file 1: **Figure S1.** Risperidone treatment does not affect tumor histopathology or lung metastatic potential. **(A)** H&E staining of tumor sections. **(B)** qPCR analysis of lung metastasis and the resulting dot plot analyzed using the Mann-Whitney test. Each dot in this plot represents one mouse. (AI 4884 kb)

Additional file 2: **Figure S2.** Risperidone treatment accelerates early lesion development of MMTV-*Wnt1* transgenic mice. **(A)** Carmine whole mount staining of mammary glands from MMTV-*Wnt1* transgenic mice. **(B)** H&E staining of mammary gland sections of MMTV-*Wnt1* mice. **(C)** pSTAT5 immunohistochemical staining of mammary gland sections of MMTV-*Wnt1* mice. (AI 22593 kb)

Additional file 3: **Figure S3.** Pimozide increases serum prolactin levels, pSTAT5 in early lesions, and β-casein, while aripiprazole does not impact pSTAT5. **(A)** Serum prolactin levels. **(B)** pSTAT5+ cells determined by immunohistochemistry analysis. **(C)** Immunohistochemistry analysis of β-casein. **(D)** pSTAT5+ cells determined by immunohistochemistry analysis. The *p* values were determined using the Mann-Whitney test. Each dot in these plots represents one mouse. (AI 6764 kb)

Additional file 4: **Figure S4.** Pimozide accelerates the development of early lesions initiated by RCAS-caErbB2 while lowering the anticancer barrier of apoptosis. **(A)** Immunohistochemical staining for the HA tag on the RCAS-ErbB2 provirus with the corresponding dot plot. **(B)** Immunofluorescence staining for Ki67 with the accompanying dot plot. **(C and D)** Immunofluorescence staining for cleaved caspase 3 **(C)** and TUNEL assay **(D)** with the accompanying dot plots. The Mann-Whitney test was used to determine the *p* values. Each dot in these plots represents one mouse. (AI 15221 kb)

Additional file 5: **Figure S5.** Effects of aripiprazole and clomipramine on biomarkers and lesion burden. **(A)** Serum prolactin levels of mice in the aripiprazole study. **(B)** Lesion burden determined by immunohistochemical staining for the HA tag on RCAS-caErbB2. **(C)** Ki67+ cells determined by immunofluorescence staining. **(D)** TUNEL+ cells. **(E)** Serum prolactin levels of mice in the clomipramine study. **(F)** Lesion burden determined by immunohistochemical staining for the HA tag on RCAS-caErbB2. The *p* values were determined using the Mann-Whitney test. Each dot in these plots represents one mouse. (AI 713 kb)

Additional file 6: **Figure S6.** Effects of genetic ablation of *STAT5a* on downstream factors of STAT5. Immunohistochemistry analysis of β-casein in caErbB2 early lesions. (AI 4570 kb)

Additional file 7: **Figure S7.** Effects of ruxolitinib on pSTAT5 and tumor latency. **(A)** Serum prolactin levels of mice. **(B)** Immunohistochemistry analysis and the accompanying dot plot for pSTAT5+ cells. The *p* values were determined using the Mann-Whitney test. Each dot in this plot represents one mouse. **(C)** Kaplan-Meier tumor-free survival curve of mice infected by RCAS-HRasQ61L. The *p* value was determined by generalized Gehan-Wilcoxon test with Rho = 1. (AI 6247 kb)
